# Multiresponsive actuators based on modified electrospun films

**DOI:** 10.1039/c7ra13384g

**Published:** 2018-03-14

**Authors:** Libiao Han, Jiang Xu, Shuai Wang, Ningyi Yuan, Jianning Ding

**Affiliations:** Jiangsu Collaborative Innovation Center of Photovoltaic Science and Engineering, Jiangsu Province Cultivation Base for State Key Laboratory of Photovoltaic Science and Technology, Changzhou University Changzhou 213164 Jiangsu China dingjn@cczu.edu.cn nyyuan@cczu.edu.cn; School of Mechanical Engineering, Changzhou University Changzhou 213164 China

## Abstract

Robot technology has made great progress over the past few decades, and has found wide use in military, industrial and service fields. In recent years, there has been a dramatic increase in the demand for soft robots. However, as a key part, the development of soft actuators capable of low-energy actuation, multi-stimulation response, and large shape deformation is still challenging. In this work, we fabricated multiresponsive actuators based on modified electrospun films. The actuators provided the largest curvatures of 0.83 cm^−1^, 0.6 cm^−1^, and 1.05 cm^−1^, stimulated by humidity, light, and electricity, respectively. Furthermore, we designed a biomimetic application—a crawling robot—which demonstrates excellent potential applications of the actuator in soft robotics, artificial muscles, and the biomimetics field.

## Abstract 2

A flexible actuator was fabricated based on modified electrospun films, which can be stimulated by humidity, light and electricity.

## Graphical abstract

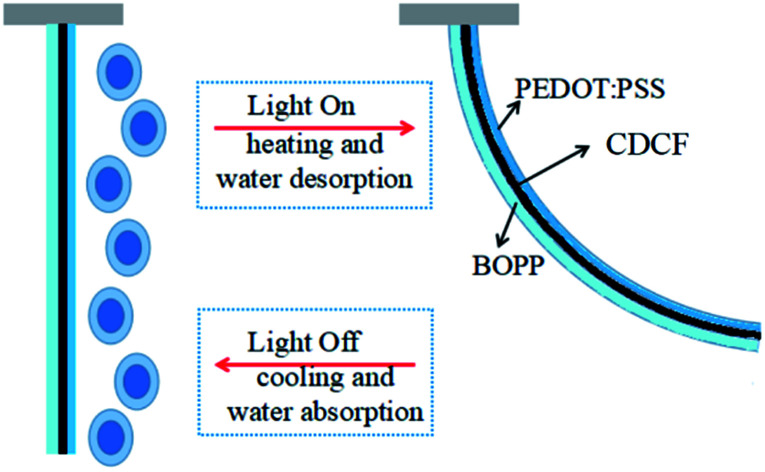

## Introduction

Robot technology has made great progress over the past few decades, and has found wide use in military, industrial and service fields.^1–3^ Traditional robots usually consist of rigid modules connected through kinematic pairs, each kinematic pair providing one or more degrees of freedom. All the movements of the moving pair combinations form the actuator at the end of the robot working space, and provide the advantage of control precision. However, the rigidity of the structure leads to poor environmental adaptability, and the movement in a complex unknown space is often unstable and lacks flexibility. Soft robots composed of flexible material with multiple degrees of freedom, simple structure, less processing cost, and no internal skeleton, can mimic biological systems with lifelike motions (*e.g.*, walkers, swimmers, rollers, grippers, tentacles),^3–6^ which can accomplish complex motions in a relatively simple manner. As a key part, soft actuators, which respond to applied external stimuli such as thermal,^7^ light,^8–11^ pressure,^12–14^ chemical,^15,16^ electric^17–20^ and humidity^21–25^ or magnetic fields,^26–28^ have been explored for biomimetic applications.^10,16,22,24^

Among various actuating structures, bilayer structures have been widely used to convert dimensional changes into bending motions.^1–6^ A bilayer system typically consists of an active layer that contracts or expands in response to an external stimulus and a passive layer that remains intact.^29–31^ Active-layer materials are mainly based on materials such as gels,^8,15,16^ paper,^20,32^ silver nanowires (AgNWs),^22,33^ carbon nanotubes (CNTs),^34–37^ and graphene and its derivatives.^9–11,17–19,38,39^ However, the manufacturing and preparation processes for some of the materials are complex, or their cost is relatively high. In addition, present actuators are mostly triggered by a single stimulus.

In this work, we fabricated multi-walled carbon nanotubes (MWCNTs)-doping cross-linked carbon nanofiber (CDCF) films using electrospinning technology,^40–42^ pre-oxidation and carbonization treatment. Electrospinning technology has become an way to make nano-fiber materials effectively, with its advantages such as simple manufacturing device, low cost of spinning, various kinds of spinning materials and controllable process. Then poly(3,4-ethylenedioxythiophene) polystyrene sulfonate (PEDOT:PSS) was drop-cast on the surface of the CDCF and combined with biaxially oriented polypropylene (BOPP) film to constitute the multiresponsive actuator. The largest bending curvatures of 0.83 cm^−1^, 0.67 cm^−1^ and 1.05 cm^−1^ stimulated by humidity, light, and electricity were obtained, respectively. Furthermore, we designed a crawling robot that could mimic the movement of inchworm. These features show that the multiresponsive actuating materials have great potential in soft robotics, artificial muscles, and biomimetics field.

## Materials and methods

**Fig. 1:**
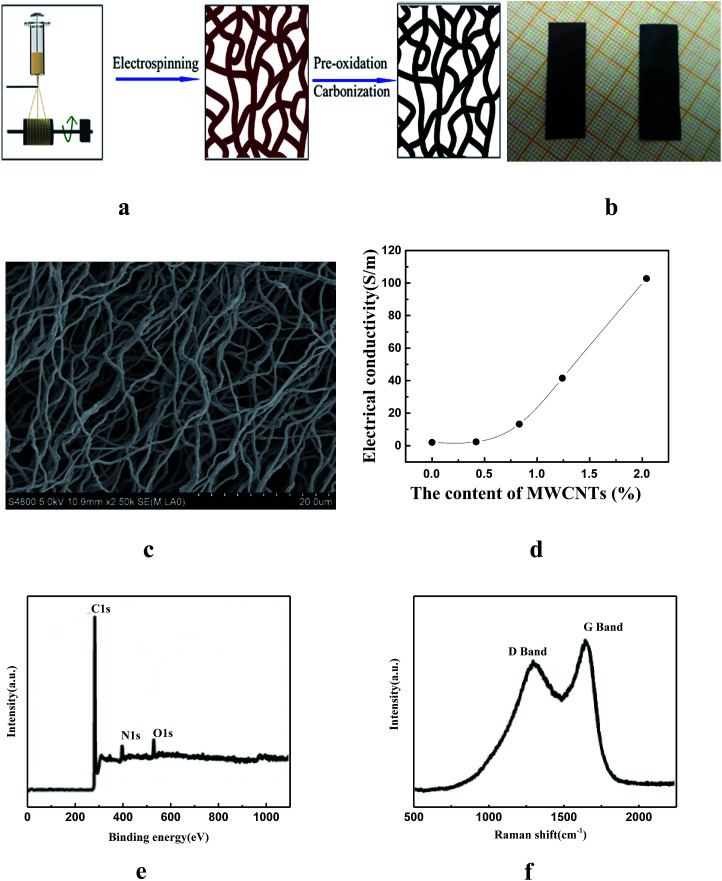
(a) Schematic of the preparation of CDCF; (b) the photograph of morphology change of the samples in preparation process (after been pre-oxidation and carbonization treatment, respectively); (c) the scanning electron microscope image of the CDCF; (d) the mass fraction of MWCNTs in the mixed solution; (e) XPS survey spectra of CDCF; (f) Raman spectra of CDCF.

The fabrication of the active layer was mainly based on electrospinning technology. Multi-walled carbon nanotubes (MWCNTs) were added to *N*,*N*-dimethylformamide (DMF) and the solutions was sonicated for three hours to ensure uniform dispersion. Then terephthalic acid (TPA), peroxyacyl nitrate (PAN), and polyvinylpyrrolidone (PVP) were added, and the mixed solution was stirred magnetically for several hours. The prepared solution was spun into a membrane with an electrospinning machine. After pre-oxidation at 270 °C in air for 2 h and carbonization at 800 °C for 1 h in Ar atmosphere, MWCNTs-doping cross-linked carbon nanofiber (CDCF) could be obtained (see the Experiment section for details), shown in Fig. 1a. Fig. 1b shows the photograph of the change in morphology of the samples in the preparation process (from left to right: after pre-oxidation and carbonization treatment, respectively). The obtained CDCF is hydrophobic. MWCNTs can improve the conductivity of the CDCF, and it is an excellent light-responsive material. Fig. 1c is the scanning electron microscope image of the CDCF, and MWCNTs are embedded in carbon nanofiber. The increase of the proportion of MWCNTs in the CDCF can increase the conductivity, as shown in Fig. 1d. However, adding too much MWCNTs made it difficult to disperse uniformly in the solution. More importantly, too much MWCNTs in the CDCF would lead to a decrease in the flexibility of the CDCF. Hence, the 2.04 wt% of MWCNTs in the mixed solution was chosen in subsequent experiments. The elemental composition and bonding configurations of CDCF were probed by X-ray photoelectron spectroscopy (XPS), as shown in Fig. 1e. Three typical peaks corresponding to the binding energies of C 1s, N 1s and O 1s are observed. Fig. 1f is Raman spectra CDCF to investigate its structure, two characteristic peaks at about 1351 and 1586 cm^−1^ correspond to the disorder-induced D band and inplane vibrational G band of carbonaceous materials, respectively. The relatively intensity of the G band is slightly higher than that of the D band, indicating the effect of MWCNTs. To improve the moisture absorption of the CDCF, after air plasma treatment, 60 μL cm^−2^ of poly(3,4-ethylenedioxythiophene) polystyrene sulfonate (PEDOT:PSS) was then drop-cast on the one side of the CDCF and dried with light heating. At this point, the active layer—CDCF coated with PEDOT:PSS—was fabricated. BOPP was chosen as the passive layer because of its large coefficient of thermal expansion (CTE). Finally, BOPP films were attached to the other side of CDCF with acrylic adhesive to form a sandwich structure, and the multi-response actuators were obtained.

## Results and discussion

### Response to humidity

**Fig. 2:**
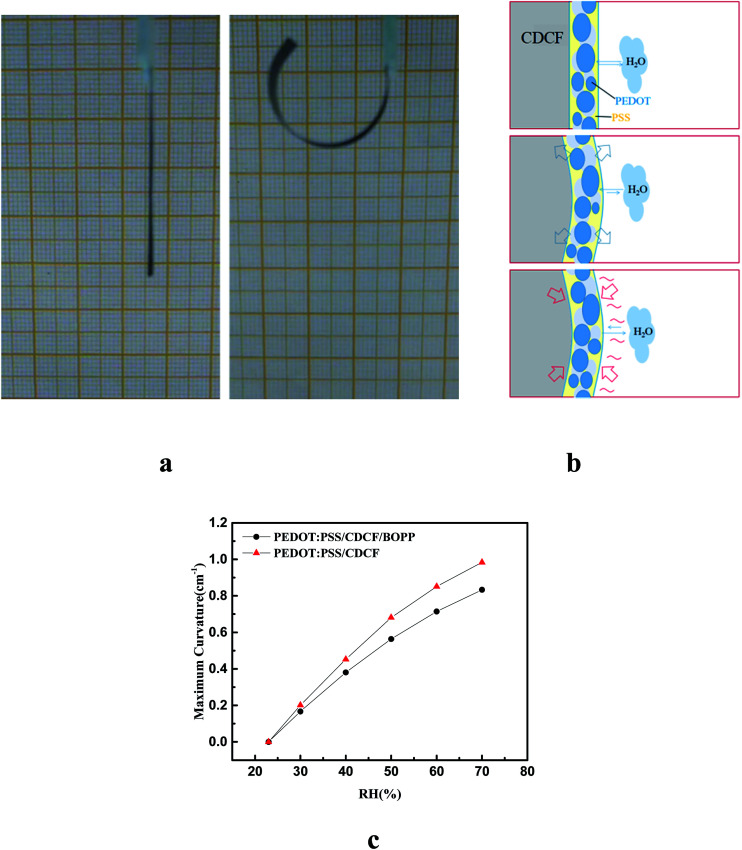
Bending performance of the actuator response to humidity. (a) The initial state, and the bent state with the maximum curvature; (b) schematic of the humidity-drive actuation mechanism; (c) curvature of the PEDOT:PSS/CDCF/BOPP actuator and the PEDOT:PSS/CDCF actuator as functions of the change of RH.

In this study, PEDOT:PSS was drop-cast on the surface of CDCF which was subjected to air plasma treatment to increase the coefficient of hygroscopic expansion (CHE). The actuator showed a large bending toward the BOPP side in a humidity atmosphere. To investigate the actuation characteristic of the PEDOT/CDCF/BOPP actuator quantitatively when driven by humidity, we constructed transparent humidity-control chamber to monitor the actuation. A 30 mm-long actuator in a flat state with one end fixed on a glass slide and suspended in atmosphere, as shown in Fig. 2a. We measured the indoor relative humidity was 23%. Then we transferred the actuator into the humidity-control chamber with 75% humidity, where the actuator showed a large bending actuation in 60 s. The curvature was up to 0.83 cm^−1^, as shown in the right panel of Fig. 2a. The actuator recovered to the flat state after being taken out of the humidity control chamber. Fig. 2b schematically shows the humidity-driven bending actuation mechanism. When the ambient humidity increases, water molecules are adsorbed on the PEDOT:PSS layer, which results in an increase in the moisture content, leading to the swelling of the PEDOT:PSS layer. On the contrary, the CDCF and BOPP film are inert to water molecules and act as passive layer, while the PEDOT:PSS layer acts as active layer. When the ambient humidity decreases, the PEDOT:PSS layer will desorb water molecules, resulting in the shrinking of the PEDOT:PSS layer, and the actuator recovers its original state slowly. A control experiment showed that without PEDOT:PSS layer the actuator exhibited no bending in response to humidity changes, which demonstrates that the large bending of the actuator is mostly attributed to the swelling of the PEDOT:PSS layer when humidity changes. We further investigated the influence of the humidity on the bending curvature. As shown in Fig. 2c, the curvature of the actuator increased from 0 to 0.83 cm ^−1^, when the RH increased from 23% to 75%. Because the BOPP and the CDCF are both hydrophobic, we investigated the performance of the PEDOT:PSS/CDCF actuator, as shown in Fig. 2c. We noticed that the performance of the bilayer actuator is little superior to that of the trilayer actuator, this can be explained by that the BOPP increased the total thickness of passive layer and limited the bending.

### Response to light

**Fig. 3:**
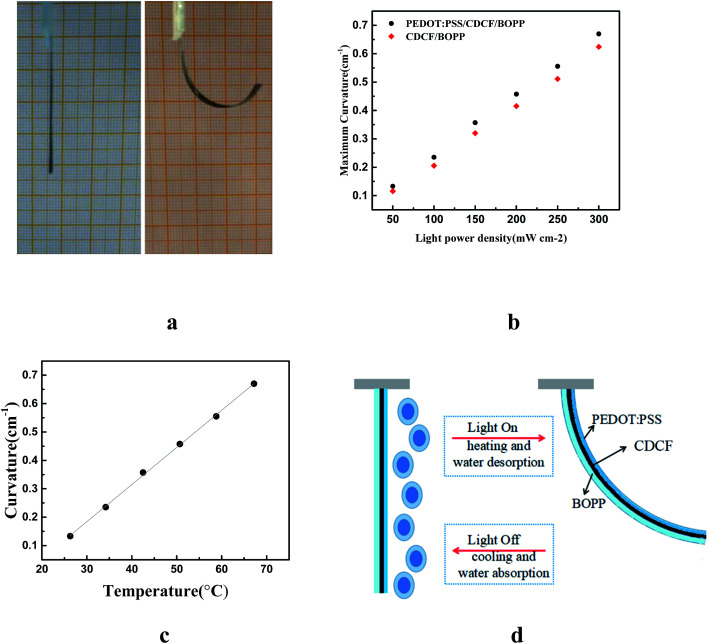
Bending performance of the trilayer actuator response to humidity. (a) The optical photo showing the actuator response to the light. (b) Curvature of the PEDOT:PSS/CDCF/BOPP actuator and the CDCF/BOPP actuator as functions of light power density (mW cm^−2^); (c) curvature of the actuator dependence of temperature; (d) schematic of the light-drive actuation mechanism.

The performance of the actuator in response to near infrared (NIR) light was investigated. A 30 mm-long actuator was fabricated at an RH of 23%. When an NIR light irradiation was on the BOPP side, the actuator bent toward the CDCF side, as shown in the right panel of Fig. 3a. Fig. 3b demonstrates the light power density dependence of the curvature and the temperature of the actuator. It can be clearly seen that a larger light power density results in a larger curvature of the actuator. With a larger light power density, there is more NIR light converting into thermal energy by CDCF layer, resulting in a higher temperature. The maximum curvature was up to 0.67 cm^−1^ with temperature of 67.2 °C after NIR light irradiation (300 mW cm^−2^) for 30 s. A control experiment was also carried out to investigate the effect of the actuator without the PEDOT:PSS layer, as shown in Fig. 3b. In order to further study the actuation mechanism of the actuator, the curvature and temperature of the actuator were measured synchronously during the actuation, as shown in Fig. 3c. There are two mechanisms together resulting in the large bending performance of the actuator, as shown in Fig. 3d. The first mechanism is the hygroexpansion effect of the active layer. When the CFDP converts the absorbed NIR light into thermal energy, the water molecules contained in the PEDOT are evaporated because of the temperature increase. Therefore, the PEDOT layer shrinks after water molecules desorb, while the BOPP is inert to humidity change, which leads to the actuator bending toward the PEDOT layer side. Second, there is a great CTE mismatch between CDCF and BOPP. CDCF is a carbon cellulose-based material, which has a small CTE, while the BOPP has a large CTE of 137 ppm K^−1^. The CDCF shows excellent absorption in the wavelength range from ultraviolet (UV) light to NIR light, while the BOPP has nearly no absorption. When the actuator is irradiated by the NIR light, the active layer absorbs the photonic energy. Then, the absorbed photonic energy is quickly converted into thermal energy, leading to a temperature increase in the active layer. Then the heat is effectively transported to BOPP. As the CTE of BOPP is much larger than that of CDCF, the larger expansion of the BOPP film results in the actuator bending toward the CDCF side with temperature increase.

### Response to electricity

**Fig. 4:**
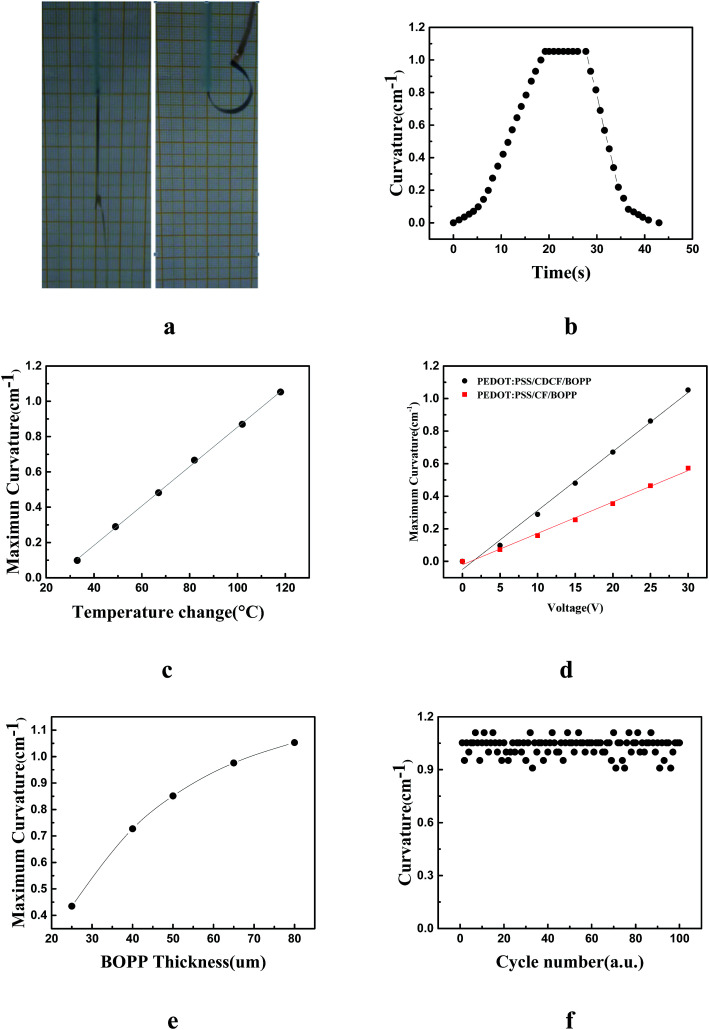
Bending performance of the actuator response to electricity. (a) The initial state, and the bent state with the maximum curvature; (b) curvature of the actuator as functions of time; (c) the generated maximum curvature *versus* the temperature change; (d) curvature of the PEDOT:PSS/CDCF/BOPP actuator and the PEDOT:PSS/CF/BOPP as functions of applied DC voltage; (e) curvature as a function of BOPP thickness under an applied voltage of 30 V DC; (f) repeatability test of the actuator with a rectangular wave voltage (0–30 V) for 100 cycles.

Furthermore, we investigated the performance of the actuator when stimulated by electricity. The fabrication process of electrical-driven PEDOT:PSS/CDCF/BOPP actuator is a little different from the humidity-driven and light-driven actuator, as copper electrodes should be embedded during the fabrication process. After the PEDOT:PSS was deposited on the one side of the CDCF, copper electrodes were pasted on the ends of the other side of the CDCF with silver paste. Then we combined the BOPP and the CDCF with acrylic adhesive. When the actuator was stimulated by electricity, obvious bending toward the active layer side was observed, as shown in Fig. 4a. The mechanism of the actuator driven by electricity is similar with light stimulation. When a DC voltage was applied, the actuator would be Joule-heated by resistance. Because of the mismatch between the CDCF and the BOPP film, the actuator will bend to the CDCF side. Meanwhile, the water molecules contained in the PEDOT:PSS are evaporated because of the temperature increase, which will intensify the bending. Upon the application a DC voltage of 30 V, the actuator reached the maximum curvature in approximately 20 s and maintained the curvature with the voltage on. When the voltage was turned off the voltage, the tip of the actuator was completely restored to initial position within 15 s when it cooled down to the room temperature. A maximum bending angle of 270° and curvature of 1.05 cm^−1^ were achieved with an input power density of 18.9 mW mm^−2^. Fig. 4b shows the bending curvature of the actuator that was acquired by an infrared camera as a function of time. Fig. 4c shows that the curvature has a linear relationship with the temperature change. The average temperature obtained from the two surfaces of the CDCF and BOPP was used here; no obvious difference in the measured temperatures was observed. The change in the curvature nearly followed the temperature change. To investigate the effect of the conductivity of the CDCF, we compared the performance of the PEDOT:PSS/CDCF/BOPP actuator and a actuator without doping MWCNTs, as shown in Fig. 4d. Furthermore, based on the analysis results, the bending curvature of the actuator is independent of the width and length of the actuator, while it is influenced by the thickness of the actuator. To simplify the problem, the thickness of the CDCF and PEDOT were fixed, while the thickness of the BOPP film was altered. We found that the thickness of the BOPP film has a pronounced influence on the bending performance of the actuator. The maximum curvature of the actuator decreased with the increase in the thickness of the BOPP film, as shown in Fig. 4e. A repeatability test of the actuator driven by electricity was also conducted. A rectangular wave voltage (0–30 V) was applied to the actuator for 100 cycles. The performance was reasonably repeatable and no obvious degradation was observed, indicating good reliability and stability, as shown in Fig. 4f. The actuator can be cut into the desired shapes and dimensions. For example, strip actuators were used for the walkers and the grippers. This simple “tape and cut” process for making the soft actuators can be readily extended to mass fabrication.

### Application of the soft actuator

**Fig. 5:**
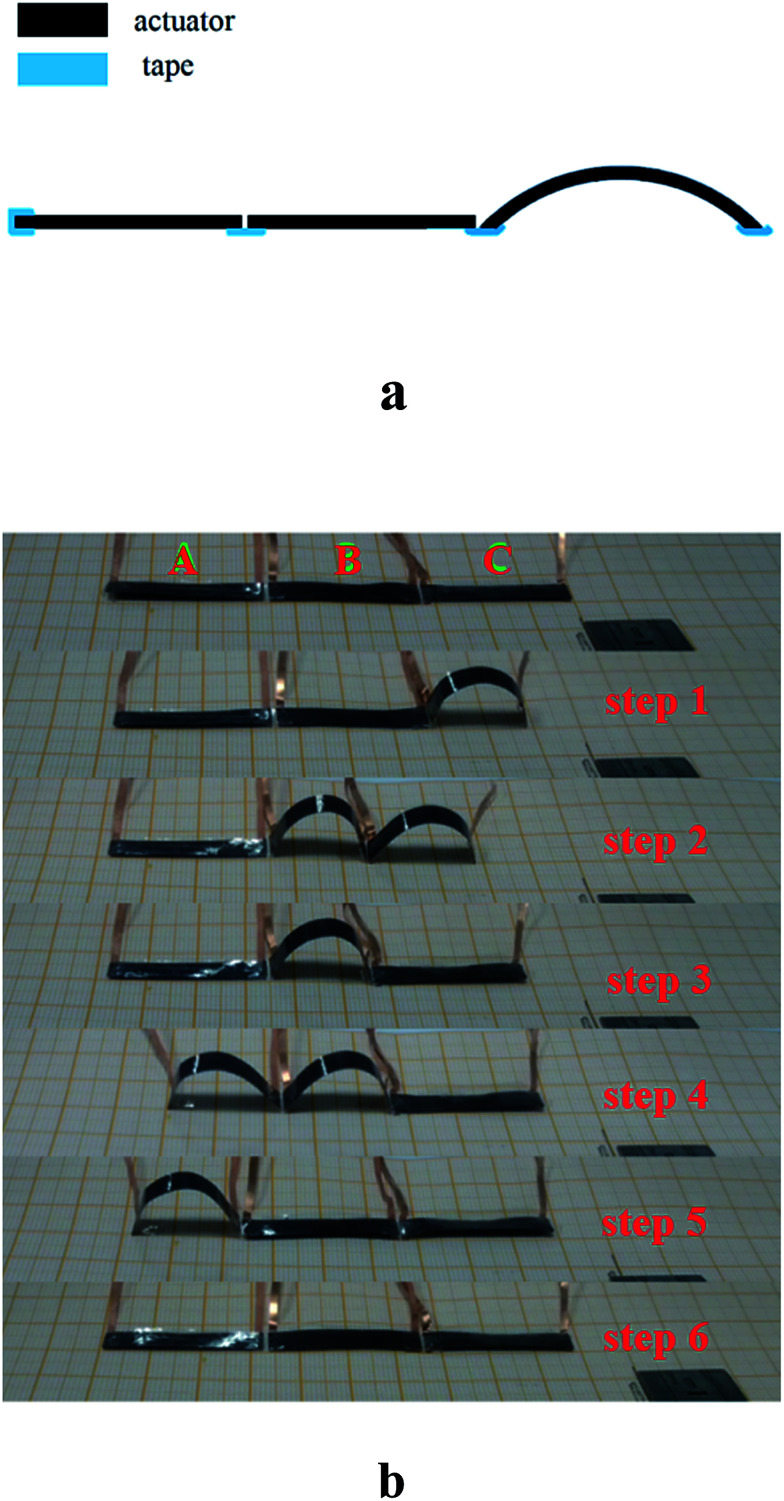
(a) The schematic of fabrication of the crawling robot; (b) optical photo of six steps of single cycle of the crawling robot.

Self walking and grabbing objects are among the basic motions of robots and are often achieved in industrial robots using electric motors and/or hydraulics with complex and rigid components. Many crawling robots based on soft actuators have been reported. Yao *et al.* reported an electrothermal actuator based on silver nanowires (AgNWs), which employed a ratchet mechanism with an asymmetrical surface structure to promote unidirectional walking motion.^33^ Chen *et al.* fabricated a super-aligned carbon nanotube/BOPP (SACNT/BOPP) composite film actuator, in which one end was tailored into a zigzag shape to realize the friction coefficient difference between the ends.^36^ However, these reported flexible actuators can only realize the unidirectional walking motion. In this work, we mimic the inchworms and designed a bidirectional motion. The crawling robot was fabricated by a simple method—connecting the three same actuators with adhesive tape, as shown in Fig. 5a. The moving direction of the crawling robot can be regulated by arranging the order of applying voltage. We defined the three actuators as A, B, and C, as shown in Fig. 5b. The single cycle of the periodic motion contains of six steps—electrifying the C, electrifying the B, deenergizing the C, electrifying the A, deenergizing the B, and deenergizing the A, and then the crawling robot can move toward the left. The mechanism is as follows. Because the three actuators are almost symmetrical and same, the normal force (*N*) acting on the end of actuators is assumed to be equal to the of gravity force (*G*). Before applying voltage on the actuator and it is flat state, the contact area between the actuator and the substrate is the PEDOT layer and the maximum static friction is *f*_1_. When the actuator is electrified, the contact area between the end of the actuator and the substrate is the additional BOPP layer and the maximum static friction acting on the end of bending actuator is *f*_2_. As the PEDOT layer surface is rougher than the BOPP surface, it would give the result of *μ*_PEDOT_ ≫ *μ*_BOPP_ (the friction coefficient of the active layer and the BOPP between the substrate respectively). Hence, the comparison between two static frictions is given by *f*_1_ = *μ*_PEDOT_*N* > *f*_2_ = *μ*_BOPP_*N*. In step one, *f*_1A_ + *f*_1B_ > *f*_2C_, and the right end of the C slide toward the left. In step two, *f*_1A_ > *f*_2B_ + *f*_2C_, and the bending C and the right end of the B slide toward the left. In step three, *f*_1A_ + *f*_2B_ > *f*_2C_, and the right end of the C slide backward. In step four, *f*_2A_ < *f*_2B_ + *f*_1C_, and the left end of the C slide toward the right. In step five, *f*_2A_ + *f*_2B_ < *f*_1C_, and the bending A and the left end of the B slide toward the left. In step six, *f*_2A_ < *f*_1B_ + *f*_1C_, the left end of the A slides toward the left, completing the entire walking process. With periodic electrical stimulus, the robot shows a continuous walking motion, which biomimetically mimics the motion model of animals such as limbless worms. On the contrary, if we apply the voltage in reverse order—electrifying the A, electrifying the B, deenergizing the A, electrifying the C, deenergizing the B, and deenergizing the C, and then the actuator can move toward the right. The self walking robot demonstrated here can benefit the development of biomimetic robots that enable autonomous motions.

## Conclusion

In this work, we prepared CDCF films using electrospinning technology, and then PEDOT:PSS was drop-cast on the one side of the CDCF. Finally BOPP was attached on the other side of the CDCF to fabricate the multiresponsive actuator. In this actuator, MWCNTs were used for improving the conductivity of the CDCF and PEDOT:PSS was used to improve the moisture absorption of the CDCF. The great mismatch in CTE and CHE between the active layer and the passive layer promised multiresponsive ability of the actuator. The largest bending curvatures of 0.83 cm^−1^, 0.67 cm^−1^ and 1.05 cm^−1^ stimulated by humidity, light, and electricity were obtained, respectively. Furthermore, we designed and fabricated a crawling robot that could realize a bidirectional motion. These features show that the multiresponsive actuators have great potential in soft robotics, artificial muscles, and biomimetics field.

## Experimental section

### Preparation of the actuator

0.5 g MWCNTs powder was added into 20.0 mL *N*,*N*-dimethylformamide (DMF) and the solution was sonicated for 3 h under ice-water condition. Then 1.0 g TPA, 1.6 g PAN and 2.4 g PVP were added into the solutions to form uniform dispersion liquid after magnetically stirring for several hours. This solution then ejected form the stainless steel capillary with a voltage of 25 kV under the feeding the feeding rate of 1.8 mL h^−1^. The distance between the capillary was 18 cm. The electrospinning precursor fiber was collected as a thin web on a rotating aluminum drum with the rotation speed of 360 rpm. After being pre-oxidation at 270 °C in air for 2 h and carbonization at 800 °C for 1 h in Ar atmosphere, CDCF can be obtained. After been air plasma treatment, 60 μL cm^−2^ of PEDOT:PSS solution was then drop-cast on the surface of CDCF and dried under light heating. Finally, the acrylic adhesive was used to combined the CDCF and BOPP films and the multi-response actuators were prepared.

### Characterization of the sandwich structure actuators

The voltage was supplied to the heaters by a power supply (Agilent 6631C) and the resulting bending angles of the trilayer actuator were recorded by using a digital camera. The temperatures of the actuators were measured in real time using a non-contact infrared camera (FLIR A655SC) placed right over the heaters or actuators. The emissivity of materials was calibrated using electrical tape over a temperature range of RT to 200 °C.

## Conflicts of interest

There are no conflicts to declare.

## Supplementary Material

## References

[cit1] Brochu P., Pei Q. (2010). Macromol. Rapid Commun..

[cit2] Hines L., Petersen K., Lum G. Z., Sitti M. (2017). Adv. Mater..

[cit3] Hu Y., Chen W. (2011). Macromol. Chem. Phys..

[cit4] Manourasa T., Vamvakaki M. (2016). Polym. Chem..

[cit5] Sellinger A. T., Wang D. H., Tan L.-S., Vaia R. A. (2010). Adv. Mater..

[cit6] Rus D., Tolley M. T. (2015). Nature.

[cit7] Janbaz S., Hedayati R., Zadpoor A. A. (2016). Mater. Horiz..

[cit8] Kim D., Lee H. S., Yoon J. (2016). Sci. Rep..

[cit9] Chen L., Weng M., Zhou P., Zhang L., Huang Z., Zhang W. (2017). Nanoscale.

[cit10] Deng J., Li J., Chen P., Fang X., Sun X., Jiang Y., Weng W., Wang B., Peng H. (2016). J. Am. Chem. Soc..

[cit11] Hu Y., Wu G., Lan T., Zhao J., Liu Y., Chen W. (2015). Adv. Mater..

[cit12] Larson C., Peele B., Li S., Robinson S., Totaro M., Beccai L., Mazzolai B., Shepherd R. (2016). Science.

[cit13] Shepherda R. F., Ilievskia F., Choia W., Morina S. A., Stokesa A. A., Mazzeoa A. D., Chena X., Wanga M., Whitesidesa G. M. (2011). Proc. Natl. Acad. Sci. U. S. A..

[cit14] Yuk H., Lin S., Ma C., Takaffoli M., Fang N. X., Zhao X. (2017). Nat. Commun..

[cit15] Wani O. M., Zeng H., Priimagi A. (2017). Nat. Commun..

[cit16] Huang C., Lv J.-a., Tian X., Wang Y., Yu Y., Liu J. (2015). Sci. Rep..

[cit17] Li W., Li F., Li H., Su M., Gao M., Li Y., Su D., Zhang X., Song Y. (2016). ACS Appl. Mater. Interfaces.

[cit18] Tsao C.-W., Guo X.-C., Hu W.-W. (2016). RSC Adv..

[cit19] Jiang Y., Hu C., Cheng H., Xu T., Zhao Y., Shao H., Qu L. (2016). ACS Nano.

[cit20] Wang D., Lu C., Zhao J., Han S., Wua M., Chen W. (2017). RSC Adv..

[cit21] Han D.-D., Zhang Y.-L., Jiang H.-B., Xia H., Feng J., Chen Q.-D., Xu H.-L., Sun H.-B. (2015). Adv. Mater..

[cit22] Amjadi M., Sitti M. (2016). ACS Nano.

[cit23] Wang D. H., McKenzie R. N., Buskohl P. R., Vaia R. A., Tan L.-S. (2016). Macromolecules.

[cit24] Mu J., Hou C., Zhu B., Wang H., Li Y., Zhang Q. (2015). Sci. Rep..

[cit25] Taccola S., Greco F., Sinibaldi E., Mondini A., Mazzolai B., Mattoliuators V. (2015). Adv. Mater..

[cit26] Miyashita S. , Guitron S., Ludersdorfer M., Sung C. R., and Rus D., IEEE International Conference on Robotics and Automation (ICRA), 2015, pp. 1490–1496

[cit27] Uh K., Yoon B., Lee C. W., Kim J.-M. (2015). ACS Appl. Mater. Interfaces.

[cit28] Gao W., Wang L., Wang X., Liu H. (2016). ACS Appl. Mater. Interfaces.

[cit29] Sun G., Zhang X., Lin R., Chen B., Zheng L., Huang X., Huang L., Huang W., Zhang H., Chen P. (2016). Adv. Electron. Mater..

[cit30] Xu F., Zhu Y. (2014). Adv. Mater..

[cit31] Wang Y., Zhu C., Pfattner R., Yan H., Jin L., Chen S., Molina-Lopez F., Lissel F., Liu J., Rabiah N. I., Chen Z., Chung J. W., Linder C., Toney M. F., Murmann B., Bao Z. (2017). Sci. Adv..

[cit32] Weng M., Zhou P., Chen L., Zhang L., Zhang W., Huang Z., Liu C., Fan S. (2016). Adv. Funct. Mater..

[cit33] Yao S., Cui J., Cui Z., Zhu Y. (2015). Nanoscale.

[cit34] Li Q., Liu C., Lin Y.-H., Liu L., Jiang K., Fan S. (2015). ACS Nano.

[cit35] Hu Y., Wang G., Tao X., Chen W. (2011). Macromol. Chem. Phys..

[cit36] Chen L., Weng M., Zhou Z., Zhou Y., Zhang L., Li J., Huang Z., Zhang W., Liu C., Fan S. (2015). ACS Nano.

[cit37] Chen L., Weng M., Zhang W., Zhou Z., Zhou Y., Xia D., Li J., Huang Z., Liu C., Fan S. (2016). Nanoscale.

[cit38] Secor E. B., Gao T. Z., Islam A. E., Rao R., Wallace S. G., Zhu J., Putz K. W., Maruyama B., Mark C. (2017). Chem. Mater..

[cit39] Bi H., Yin K., Xie X., Zhou Y., Wan S., Banhart F., Sun L. (2013). Nanoscale.

[cit40] Cheng Y., Huang L., Xiao X., Yao B., Yuan L., Li T., Hu Z., Wang B., Wan J., Zhou J. (2015). Nano Energy.

[cit41] Kim M., Kim Y., Lee K. M., Jeong S. Y., Lee E., baeck S. H., Shim S. E. (2016). Carbon.

[cit42] Seok S., Onal C. D., Cho K.-J., Wood R. J., Rus D., Kim S. (2013). IEEE/ASME Transactions on Mechatronics.

